# Supplementary material for: ‘Survival-driven’ or ‘policy-driven’—research on the motivation and economic consequences of the mixed-ownership reform of state-owned enterprises
Source: PLoS One. 2026 Jan 5;21(1):e0339970. doi: 10.1371/journal.pone.0339970 (PMC12768374; doi:10.1371/journal.pone.0339970)
Supplement: Attachment — Submitted filename: Reviewers Comment.docx [file pone.0339970.s002.docx]

The article presents an engaging argument in exploring whether or not the resource endowments of mixed-ownership reforms are the root causes of the different economic consequences of mixed-ownership reform.

However, highlighted below are some of the comments that the author should consider to improve the scholarship of the article:

About 66% of the work cited in this paper were articles published well over five years ago. This does not show current scholarship in the field. The research could be enhanced by integrating more recent studies and data, especially those published in the last five years.

Also, some of your sentences are unnecessarily lengthy, which obscures their flow and clarity of the texts. The study will be improved if you present your arguments in sizable and digestible bits. For instance, in the last paragraph under the Research Background section, the sentence starting with “First, starting from the perspective of the motivation…” can be broken down for easier comprehension.

Moreso, in the first paragraph of the Research Background section, the author introduced the concept of mixed-ownership reform, followed by the following phrase (hereinafter referred to as “mixed reform”). This by extension means that going forward, “mixed-reform” will be used in place of mixed-ownership reform. Regrettably, the author continued using the former over the latter. This unfortunately does not conform to the rules of academic writing.

Furthermore, the H1 hypothesis captured under the subheadings “The breadth of mixed-ownership reform and its effects” was adequately crafted. However, attaching the decision rules (i.e, that is, the greater the breath of the mixed-ownership reform and the more type of equity introduced, the greater the effect of the mixed-ownership reform) to the hypothesis at this stage makes it redundant. Consider moving the decision rule to the analysis section of the study to improve clarity. The same also applies to the H2 hypothesis.

Lastly, your Sample Selection and Data Source section lacks clarity and scientific rigor. Are the areas under this section labelled (1) to (4) the exclusion criteria of the literature used for this study or another set of variables altogether? Following this, what do you mean by “Ultimately, 4,124 firm-year observations are obtained”? What is the rationale/justification for selecting the state-owned listed enterprises between 2013 to 2023 as the sample size?

The utility of this study as a framework for the advancement of scientific knowledge will be impoved after addressing the methodological and structural issues raised above.
