# Supplementary material for: ‘Survival-driven’ or ‘policy-driven’—research on the motivation and economic consequences of the mixed-ownership reform of state-owned enterprises
Source: PLoS One. 2026 Jan 5;21(1):e0339970. doi: 10.1371/journal.pone.0339970 (PMC12768374; doi:10.1371/journal.pone.0339970)
Supplement: Attachment — Submitted filename: Response to Reviewers.docx [file pone.0339970.s003.docx]

Dear Editor,

I sincerely appreciate the opportunity to revise our manuscript entitled "'Survival Needs' or' Policy Promotion '-- Research on the Motivation and Economic Consequences of the Mixed Ownership Reform of State owned Enterprises" . I am grateful to the reviewers for their insightful comments, which have significantly improved the quality of our work.Below is a summary of major revisions.

Reviewer #1

**Comments（1）:About 66% of the work cited in this paper were articles published well over five years ago. This does not show current scholarship in the field. The research could be enhanced by integrating more recent studies and data, especially those published in the last five years.**

Response： I acknowledge that approximately 66% of the cited literature was published over five years ago. This is partly because our study builds upon foundational theories and early empirical evidence that remain highly relevant to our framework. However, I fully agree that integrating recent scholarship would strengthen the paper. In the revised manuscript, A total of 21 references (specifically #4, #6-15, #17-24, #27, and #31) have been updated to studies published within the last two years. After this revision, 87.5% of the references in the bibliography now consist of recent literature (2024–2025).

**Comments（2）:Also, some of your sentences are unnecessarily lengthy, which obscures their flow and clarity of the texts. The study will be improved if you present your arguments in sizable and digestible bits. For instance, in the last paragraph under the Research Background section, the sentence starting with “First, starting from the perspective of the motivation…” can be broken down for easier comprehension.**

Response：

Breaking down lengthy sentences (e.g., the highlighted paragraph in the Research Background section), splitting complex ideas into shorter, more digestible statements while preserving their logical flow.

Streamlining redundant phrasing across theoretical and methodological discussions, ensuring conciseness without sacrificing rigor.

Adding clear transitional markers (e.g., "First," "In contrast," "Consequently") to enhance coherence.

All modifications are highlighted in the tracked-changes version of the manuscript ( page 3-4). We believe these edits significantly improve the accessibility of our arguments while maintaining their academic precision.

**Comments（3）:Moreso, in the first paragraph of the Research Background section, the author introduced the concept of mixed-ownership reform, followed by the following phrase (hereinafter referred to as “mixed reform”). This by extension means that going forward, “mixed-reform” will be used in place of mixed-ownership reform. Regrettably, the author continued using the former over the latter. This unfortunately does not conform to the rules of academic writing.**

Response：

Initial Occurrence：Standardized the first mention as "mixed-ownership reform (hereinafter referred to as 'MOR')" in the Abstract section (Line 5, Page 1).

Systematic Replacement：Consistently used "MOR" thereafter throughout the manuscript, verified by Word's "Find & Replace" function.

All modifications are highlighted in the tracked-changes version. We fully acknowledge the importance of terminological precision in scholarly writing and will maintain stricter standards in future works.

**Comments（4）:Furthermore, the H1 hypothesis captured under the subheadings “The breadth of mixed-ownership reform and its effects” was adequately crafted. However, attaching the decision rules (i.e, that is, the greater the breath of the mixed-ownership reform and the more type of equity introduced, the greater the effect of the mixed-ownership reform) to the hypothesis at this stage makes it redundant. Consider moving the decision rule to the analysis section of the study to improve clarity. The same also applies to the H2 hypothesis.**

Response：

Hypothesis Refinement: Removed the redundant decision rules from both H1 ("breadth of mixed reform") and H2 (Page 6-7), retaining only core hypothetical relationships.

Structural Reorganization: Relocated the decision rules ("diversity of equity types positively correlates with reform effectiveness") to Section 4 (Empirical result analysis) as empirical testing criteria.

All changes are tracked in the revised manuscript . We believe this modification enhances methodological rigor while improving readability.

**Comments（5）:CommentsLastly, your Sample Selection and Data Source section lacks clarity and scientific rigor. Are the areas under this section labelled (1) to (4) the exclusion criteria of the literature used for this study or another set of variables altogether? Following this, what do you mean by “Ultimately, 4,124 firm-year observations are obtained”? What is the rationale/justification for selecting the state-owned listed enterprises between 2013 to 2023 as the sample size?**

Response：

I deeply appreciate the reviewer's rigorous scrutiny on sampling methodology. Substantive revisions have been made on pp.9-10:

(1)The time frame is anchored in China’s policy timeline and research feasibility:first, the 2013 Third Plenary Session of the 18th CPC Central Committee launched the new round of mixed-ownership reform for state-owned enterprises (SOEs), making 2013 a pivotal starting point for analyzing policy-driven reform effects .Second,the 2013–2023 period captures the full implementation phase of MOR, allowing sufficient time to observe long-term economic consequences (e.g., governance optimization, performance improvement) .Finally, Post-2013 data benefits from improved disclosure standards of listed SOEs, while 2023 serves as the latest feasible endpoint for comprehensive data collection.

(2) Exclude samples from the financial industry.Financial enterprises are omitted due to their unique regulatory environments and accounting standards, which differ significantly from non-financial sectors and may confound empirical results;

(3) Remove observations of enterprises that were classified as ST or *ST during the sample period.Firms classified as ST (Special Treatment) or *ST (risk of delisting) during the sample period are excluded, as their financial distress and operational anomalies could distort the evaluation of mixed-ownership reform effects .;

(4) Delete samples with missing values for key variables.Samples lacking data on core variables (e.g., ownership structure, financial performance) are excluded to maintain data integrity and analytical reliability ;

(5) All continuous variables (e.g., ROA, Tobin’s Q) are winsorized at the 1% and 99% tails to reduce the influence of extreme values on regression results .

(6)The initial sample for the mixed-ownership reform of state-owned enterprises (SOEs) from 2013 to 2023 comprised 5,923 observations. After excluding financial enterprises, the sample size was reduced to 5,074. Further removal of ST/*ST enterprises led to 4,190 observations, and deletion of firms with missing key variables resulted in a final sample of 4,124.The balanced panel dataset consists of 4,124 firm-year observations, derived from 810 listed companies over a 11-year observation period.

All changes are tracked in the revised manuscript .

**Reviewer #2: The research paper’s abstract is not clearly written and does not impress because of several disadvantages. It does not discuss the techniques used to analyse data, nor the sources of that data, so its analysis seems flawed. Some important issues are the lack of financial and ST/ST firms, not defining “breadth” and “depth” in reforms and using data manually that was not verified by any procedures. Also, the study does not provide enough information about the sample companies used, as well as how control groups are selected in the quasi-experimental method. The conclusions are described using general words and do not suggest any practical steps. For improvement, the abstract ought to be clearer, introduce major analytics, explain how science was conducted, restate results and advise on policy matters. It is important to explain why certain samples were not included, to identify main metrics, to prove that the data is reliable, to share full information about the samples and to outline how control groups were used in the study.**

Response：

Thank you very much for your detailed and constructive comments on our manuscript. We sincerely appreciate your time and effort in reviewing our work, and we fully acknowledge the issues you raised regarding the abstract. Your feedback has provided us with clear directions for improvement, and we have made comprehensive revisions to address these concerns.

1. Data Analysis Techniques and Data Sources

You pointed out that the original abstract did not discuss the data analysis techniques and data sources, making the analysis seem flawed. In the revised abstract, we have explicitly stated that we adopted a rigorous quasi-experimental research design. The data is sourced from authoritative databases, including the China Stock Market & Accounting Research (CSMAR) database and the Wind database. We also collected data manually and cross-verified it with company annual reports and regulatory filings to ensure its reliability. Regarding the analysis methods, we employed fixed-effects regression models for panel data analysis . These details have been clearly presented in the new version of the abstract, enabling readers to have a better understanding of our research methodology.

2. Key Elements Missing

Exclusion of Financial and ST/ST Firms: I excluded financial and ST/ST firms from our sample because financial firms have unique business models and regulatory environments, which may introduce confounding factors to our research. ST/ST firms, on the other hand, are in a state of financial distress, and their data may distort the relationship between mixed-ownership reform and reform outcomes. This exclusion criterion has been clearly explained in the revised abstract.

Definition of “Breadth” and “Depth”: I now provide clear operational definitions for “breadth” and “depth” in the reform. “Breadth” is defined as the number of distinct shareholder types participating in the reform, while “depth” is defined as the proportion of non-state shares in the total equity structure. These definitions are now included in the abstract to ensure clarity.

Data Verification: As mentioned above, I have described in detail the data verification process in the revised abstract. The manual data was carefully cross-checked with multiple reliable sources to ensure its accuracy and reliability.

3. Insufficient Sample Information

In the original abstract, the information about sample companies and the selection of control groups was lacking. In the revised version, I have elaborated on the sample selection process. The sample companies were selected based on strict criteria, including industry representativeness, continuous operation for at least three years during the study period, and full compliance with regulatory requirements.

4. Limitations of Conclusions

I agree that the original conclusions were too general and lacked practical steps. In the revised abstract, I have proposed several specific and actionable policy recommendations based on our research findings. For example, I suggest that regulatory authorities should strengthen supervision over related transactions between non-state and state shareholders to reduce agency risks. Policy initiatives promoting mixed-ownership reform should prioritize the introduction of diverse non-state shareholders to increase the breadth of reform. Additionally, targeted policy support is needed for SOEs in less marketized regions to optimize the utilization of diversified equity structures. These policy recommendations make our conclusions more practical and valuable.

### **Statement on the Role of Funders**

The funders had no role in study design, data collection and analysis, decision to publish, or preparation of the manuscript.

The formatting of the article has also been revised according to the requirements.
