# Supplementary material for: ‘Survival-driven’ or ‘policy-driven’—research on the motivation and economic consequences of the mixed-ownership reform of state-owned enterprises
Source: PLoS One. 2026 Jan 5;21(1):e0339970. doi: 10.1371/journal.pone.0339970 (PMC12768374; doi:10.1371/journal.pone.0339970)
Supplement: Attachment — Submitted filename: Final Reviewers Comment.docx [file pone.0339970.s004.docx]

Dear Editor,

I have carefully gone through the manuscript titled “Survival-Driven or Policy-Driven – Research on the Motivation and Economic Consequences of the Mixed Ownership Reform of State-owned Enterprises” by Dr. Xiuting Wang. I must admit that the author has adequately addressed all my previous comments and made the requested changes. I am satisfied with the revisions and therefore recommend the manuscript for publication.

Thank you.
