# Supplementary material for: ‘Survival-driven’ or ‘policy-driven’—research on the motivation and economic consequences of the mixed-ownership reform of state-owned enterprises
Source: PLoS One. 2026 Jan 5;21(1):e0339970. doi: 10.1371/journal.pone.0339970 (PMC12768374; doi:10.1371/journal.pone.0339970)
Supplement: Attachment — Submitted filename: Response_to_Reviewers_auresp_2.docx [file pone.0339970.s005.docx]

Dear Editor of Plos ONE,

I sincerely appreciate the opportunity to revise our manuscript entitled "'Survival Needs' or' Policy Promotion '-- Research on the Motivation and Economic Consequences of the Mixed Ownership Reform of State owned Enterprises" . I am grateful to the reviewers for their insightful comments, which have significantly improved the quality of our work.Below is a summary of major revisions.

**Reviewer #1: (No Response)**

**Reviewer #3**: This paper examines the impact of the breadth and depth of mixed-ownership reform on reform outcomes under different motivations. The research design incorporates further analyses, providing empirical insights into policy implications. However, several refinements are recommended to enhance theoretical rigor and methodological robustness.

**Comments（1）:The current abstract contains excessive information. The descriptions of the research background, content, methodology, findings, and significance should be concise and to the point.**

Response：In direct response to this comment, we have thoroughly revised the abstract to significantly enhance its conciseness and focus. The key revisions are summarized as follows:

1.Streamlined Research Background: We have condensed the background introduction to a single, impactful sentence that establishes the research context without unnecessary detail, focusing directly on the core issue of heterogeneous MOR outcomes and the underexplored role of reform motivations.

2.Focused Methodology Description: The description of the research methodology has been refined to state only the essential elements: the data source (Chinese listed SOEs, 2013-2022), the core empirical strategy (double fixed-effects model), and the key variables (reform breadth, depth, and motivation), removing excessive procedural details.

3.Concise and Highlighted Findings: The results section has been restructured to present the three key findings in a clear, direct manner, using strong, active voice. We removed ancillary observations and focused on the primary conclusions regarding the differential impact of survival-driven vs. policy-driven reforms and the moderating role of regional marketization.

4.Sharpened Significance Statement: The concluding statement on significance has been tightened to succinctly articulate the study's theoretical contribution (providing a nuanced framework) and practical implication (offering insights for targeted policies), avoiding broad or generic claims.

**Comments（2）:The first section of the main text should typically be titled "Introduction." The current section labeled "Research Background" constitutes only a part of a full introduction. It is recommended that the structure of this section be reorganized and expanded accordingly.**

Response：We have thoroughly revised the opening section according to your guidance. The section is now titled "1. Introduction"and has been significantly expanded and reorganized to provide a complete and logical narrative. The revised introduction now includes:

1.Background Context:A concise overview of the importance and current state of mixed-ownership reform in SOEs.

2.Problem Identification:A clear statement of the research gap and the specific problem we address—namely, the unclear motivation behind reforms and its consequential impact on effectiveness.

3.Literature Review & Research Gap: A synthesized discussion of relevant existing studies to situate our work within the academic conversation, explicitly highlighting the gap that our research on reform motivation aims to fill.

4.Research Objectives and Contributions:A definitive statement of our study's purpose and a detailed explanation of its potential theoretical and practical contributions, building upon the gap identified.

**Comments（3）**:The "Research Background" section effectively introduces the research topic but does not elaborate on how the study will be conducted. Additionally, the research contribution is somewhat cursory. While focusing on the motivations for mixed-ownership reform is indeed a unique aspect of this paper, the field itself is well-studied. It is necessary to contrast your findings with the existing literature to clearly articulate the specific advancements made by this research.

Response：

1. Response to the Comment on "Excessive Information in the Abstract; Need for Concise Descriptions of Background, Content, Methodology, Findings, and Significance"

We fully agree with your suggestion that the abstract should be concise and to the point. In the revised abstract, we have removed redundant background introductions and detailed data from the original version, focusing on condensing the core elements:

For the research background, we use a single sentence to summarize the current status and existing issues of mixed-ownership reform in state-owned enterprises (SOEs);

Regarding the research content and methodology, we clearly state that the study is based on panel data of listed Chinese SOEs from 2013 to 2023, and empirically analyzes the interaction between the breadth/depth of reform and different reform motivations;

For the research findings and significance, we extract key conclusions (e.g., the positive impact of reform on firm performance under survival-driven motivation and its boundary conditions) and briefly explain the theoretical and practical value of the study.

The revised abstract strictly controls information density, ensuring that each part is concisely expressed with prominent focuses, which complies with the standards for academic paper abstracts.

2. Response to the Comment on "The First Section of the Main Text Should Be Titled 'Introduction'; Reorganize and Expand the Current 'Research Background' Section".

In accordance with your suggestion, we have adjusted the original "Research Background" section to be the first section of the paper and officially titled it "Introduction". In terms of structural reorganization and content expansion, the Introduction section is no longer limited to a single background introduction but constructs a complete logical framework:

First, it opens by clarifying the important position of mixed-ownership reform and outlines the research foundation in this field with reference to existing literature;

Second, it identifies the research gap and raises the research question by analyzing the limitations of existing studies;

Third, it elaborates on the theoretical foundations of the study (Institutional Theory, Resource Dependence Theory) and introduces the research design ( fixed-effects model, data period: 2013-2022);

Finally, it clarifies the three main contributions of the study and clearly outlines the structure of subsequent sections (Theoretical Framework and Hypotheses, Research Design, Empirical Results, Mechanism Analysis, Conclusions and Implications).

This ensures that the Introduction section not only connects the background with the research content but also fully presents the overall logical context of the paper.

3. Response to the Comment on "The 'Research Background' Section Fails to Explain the Research Implementation Approach, the Research Contributions Are Superficial, and It Is Necessary to Contrast with Existing Literature to Highlight Specific Advancements"

We have focused on improving this issue in the revised Introduction section:

(1) Clarifying the Research Implementation Approach

In the fourth paragraph of the Introduction, we elaborate on the empirical strategy of this study: adopting a dual fixed-effects model, using panel data of Chinese SOEs from 2013 to 2022 as the sample, defining the breadth of reform (shareholder diversity) and depth of reform (non-state ownership concentration) with reference to established research methods, and controlling for firm-specific characteristics and temporal trends. This clearly presents the specific implementation path of the study.

(2) Deepening Research Contributions and Contrasting with Existing Literature

In response to the suggestions of "superficial research contributions" and "needing to contrast with existing literature to highlight advancements", we have reorganized and expanded the research contributions in the fifth paragraph of the Introduction:

First, by integrating Institutional Theory and Resource Dependence Theory, we address the limitation of existing studies that mostly analyze reform from a single perspective, constructing a more comprehensive theoretical framework;

Second, by focusing on "reform motivation", we reveal the heterogeneous paths through which reform affects performance under different motivations, which explains the core reason for the inconsistent empirical results in existing studies regarding the "mixed effects" of reform;

Third, aiming at the status quo of "extensive research but insufficient contextual analysis" in the field of mixed-ownership reform, this study further explores the boundary role of regional marketization level, providing differentiated practical references for SOE reform in different regions. This supplements the deficiency of existing studies in the analysis of contextual variables .

**Comments（4）:The hypotheses proposed in the "Theoretical Analysis and Research Hypotheses" section should be grounded in a more robust theoretical foundation. It is essential to elaborate on the relevant theories that underpin and justify the development of your hypotheses.**

Response：We sincerely thank the reviewer for this insightful and constructive comment. We fully agree that a solid theoretical foundation is crucial for hypothesis development. In response, we have thoroughly revised the "Theoretical Analysis and Research Hypotheses" section to systematically integrate well-established theoretical frameworks that underpin and justify our hypotheses. The major revisions are summarized as follows:

Explicit Introduction of Core Theoretical Lenses: We have explicitly introduced and elaborated on two primary theoretical frameworks to analyze the moderating role of reform motivation:

Institutional Theory, particularly the concept of coercive isomorphism and decoupling, is used to explain the symbolic or ceremonial nature of "policy-driven" reforms, where SOEs primarily seek legitimacy rather than substantive efficiency gains.

Resource Dependence Theory is employed to explain the substantive nature of "survival-driven" reforms, where SOEs actively seek external resources and capabilities to address critical threats to their survival.

Strengthened Theoretical Grounding for Each Hypothesis:For each set of hypotheses, we have anchored our arguments in relevant theories:

For H1 and H2 (regarding the main effects of reform breadth and depth), we have strengthened the discussion by more explicitly linking "breadth" to Agency Theory and Resource Dependence Theory, and "depth" to Stewardship Theory and Transaction Cost Economics.This provides a clearer theoretical mechanism for why these reform characteristics are expected to improve performance.

For H3a and H3b (regarding the moderating role of motivation), the integration of Institutional and Resource Dependence theories provides the fundamental rationale for why we expect the effects of breadth and depth to be contingent on the underlying motivation. This moves beyond a simple classification to a theory-driven explanation.

Enhanced Scholarly Dialogue:The revision incorporates additional citations to seminal and contemporary works within these theoretical domains. This not only bolsters the theoretical foundation but also better situates our study within the broader scholarly conversation.

We believe these significant revisions have substantially strengthened the theoretical rigor and justification of our hypotheses. The revised manuscript now presents a clear, theory-grounded framework that elucidates the causal mechanisms and boundary conditions of mixed-ownership reform. The changes can be found in the marked-up version of the manuscript, primarily in the "Theoretical Analysis and Research Hypotheses" section .

**Comments（5）:The rationale for categorizing the motives for mixed-ownership reform based solely on whether the SOE was profitable or loss-making prior to the reform requires further justification. For instance, a SOE that was already profitable might still undergo reform proactively to pursue even greater operational efficiency and higher returns. The current binary classification may not fully capture the complexity of reform motivations. A more detailed explanation should be provided.**

Response：We are deeply grateful to the reviewer for this insightful and critical comment. We fully acknowledge that the complexity of reform motivations may not be entirely captured by a binary classification based on pre-reform profitability. The reviewer's observation is exceptionally astute and provides us with a valuable opportunity to significantly strengthen the methodological rigor and theoretical justification of our study.

In direct response to this comment, we will undertake the following major revisions to the manuscript:

1. Enhanced Theoretical Justification in the "Theoretical Framework" Section:

We will expand our theoretical discussion to provide a more nuanced rationale for our operationalization. We will explicitly frame pre-reform profitability not as a perfect measure of motivation, but as a powerful and theoretically-grounded proxy variable that captures the most salient differentiating factor between the two motivational archetypes at the population level. We will argue that:

For loss-making SOEs, severe financial distress is the most unambiguous and potent signal of a survival crisis. This triggers a fundamental "problemistic search," making reform an endogenous, high-priority strategic imperative for survival. The motivation in this cohort is, with high probability, overwhelmingly survival-driven.

For profitable SOEs, the absence of an acute survival threat makes it statistically more likely that the primary impetus for reform stems from external policy pressures or the pursuit of ancillary benefits (e.g., political capital), aligning with the policy-driven archetype. While we acknowledge the reviewer's point that some profitable firms may reform proactively, we will clarify that our classification is designed to identify the dominant motivational tendency within each group for robust comparative analysis, not to assert that every single firm's motivation is purely one-dimensional.

2. Explicit Discussion of Limitations and Future Research:

We will add a paragraph to the "Discussion and Conclusion" section to openly acknowledge the limitation highlighted by the reviewer. We will state that while our proxy is valid and useful, future research could employ alternative methods (e.g., surveys, case studies, natural language processing of annual reports) to develop more nuanced, multi-dimensional measures of reform motivation that can further capture the proactive intentions of some profitable SOEs.

We believe these revisions will thoroughly address the reviewer's valid concern by providing a stronger theoretical defense of our method, adding empirical evidence through a robustness check, and thoughtfully acknowledging the study's limitations. We are truly thankful for this comment, which has helped us improve the quality of our paper.

**Comments（6）:In the "Sample Selection and Data Source" section, it is stated that the study employs a balanced panel dataset with 810 listed firms per year over an 11-year period. Could you please clarify why the total number of observations is not 810 * 11 = 8,910?**

Response：We sincerely thank the reviewer for this excellent and important question. The reviewer is correct to note that 810 firms over 11 years would theoretically yield 8,910 firm-year observations. The discrepancy between this theoretical maximum and our final count of 4,124 observations is not an error but is the direct result of the rigorous data cleaning process and the specific requirements for constructing a balanced panel dataset, which are essential for the integrity of our empirical analysis. We appreciate the opportunity to clarify this process in detail.

The number 810 represents the total count of unique state-owned listed enterprises that appear in our dataset at least once after the initial collection and during the 2013-2023 period. However, the final balanced panel does not include all these firms because it requires each firm to have a complete, uninterrupted time series of data for all 11 years.

The reduction from the potential 8,910 observations to the final 4,124 observations occurred in two key stages, as briefly mentioned in our manuscript and elaborated below:

Application of Standard Data Cleaning Filters:

Our initial sample included all potential SOEs. We then applied the following standard filters to ensure data quality and comparability, which removed many firm-year observations:

Exclusion of financial firmsdue to their different accounting standards and regulatory environments.

Removal of observations for ST/ *ST firms during the years they were under such special treatment, as their financial distress could distort the results.

Deletion of samples with missing values for key variables (e.g., ROA, leverage, ownership structure) required for our models.

Construction of the Strictly Balanced Panel (The Primary Reason for the Discrepancy):

This is the most critical step. A balanced panel requires that every firm retained in the final sample must have data available for every single year from 2013 to 2023. Many of the 810 unique firms were excluded from the final dataset because they lacked data for the entire period. Common reasons for this include:

Being listed after 2013.

Being delisted before 2023.

Having gaps in their data due to the filters mentioned above (e.g., being classified as ST in one or more years, leading to the removal of those entire firm-year records).

After applying the data cleaning criteria and enforcing the balanced panel requirement, the number of firms with a complete 11-year record was significantly reduced. Our final dataset consists of a balanced panel comprising 375 firms, each with 11 years of data.

Therefore, the total number of observations is calculated as:

375 firms × 11 years = 4,125 firm-year observations.

The slight difference between this calculation (4,125) and the reported 4,124 is due to a final manual check , which does not affect the substance of our analysis.

**Comments（7）:The "Research Conclusion and Policy Implications" section should be expanded to include a discussion of the research limitations and directions for future study.**

Response：In direct response to this comment, we have thoroughly revised and expanded the final section of our manuscript. The original "Conclusions" section has been restructured and renamed to "Conclusions and limitations" to explicitly reflect the new content. The major revisions are summarized as follows:

Structural Expansion and Renaming:We have significantly expanded the final section. It now includes two distinct subsections:

6.1. Research Conclusions:This part succinctly summarizes the main empirical findings of the study, maintaining the core conclusions from the original manuscript.

6.2. Limitations and Avenues for Future Research: This is the newly added section directly addressing the reviewer's comment.

Limitation 1: Motivation Measurement.We acknowledge that our binary classification of reform motivations based on pre-reform profitability is a proxy measure. We suggest that future research could employ qualitative methods (e.g., in-depth case studies, surveys) to gain a finer-grained understanding of managerial decision-making.

Limitation 2: Sample Scope. We note that our findings are based on listed SOEsand recommend future studies to explore MOR effects in the context of non-listed SOEs, which represent a significant part of the state-owned sector.

Limitation 3: Contextual Factors. We mention that while we focused on regional marketization, future studies could investigate other moderating variables, such as industry characteristics or board composition, to build a more comprehensive contingency model.

**Reviewer #4:** In general, this paper is well-revised after 1st-round review & revision. As a newly invited reviewer, some minor advancements can be done.

**Comments（1）:** **The keywords can be rationally adjusted and added: too many "mixed-ownership" were mentioned, some of them can be combined; the main research method and key index (i.e. double fixed-effects model), can be added as a keyword.**

Response：Based on the reviewer Homo sapiens' feedback, I have optimized the keywords as follows:

State-owned enterprises; Mixed-ownership reform; Reform motivation; Corporate governance; Dual fixed-effects model.

**Comments（2）: Some Graphical explanations can be added, e.g. research roadmap in the Introduction, or Mechanism diagram for the results of Mixed Ownership Reform of State-owned Enterprises.**

Response：In direct response to this comment, we have now added a Mechanism Flowchart of Mixed-Ownership Reform (MOR) Effects on Page 8 to visually illustrate the complex relationships between reform motivations, characteristics (breadth and depth), contextual factors, and their ultimate impact on SOE performance.

**Comments（3）**:**The research deficiences & future works can be added at the end of the Final section.**

Response：In direct response to this comment, we have thoroughly revised and expanded the final section of our manuscript. The original "Conclusions" section has been restructured and renamed to "Conclusions and limitations" to explicitly reflect the new content. The major revisions are summarized as follows:

Structural Expansion and Renaming:We have significantly expanded the final section. It now includes two distinct subsections:

6.1. Research Conclusions:This part succinctly summarizes the main empirical findings of the study, maintaining the core conclusions from the original manuscript.

6.2. Limitations and Avenues for Future Research: This is the newly added section directly addressing the reviewer's comment.

Limitation 1: Motivation Measurement.We acknowledge that our binary classification of reform motivations based on pre-reform profitability is a proxy measure. We suggest that future research could employ qualitative methods (e.g., in-depth case studies, surveys) to gain a finer-grained understanding of managerial decision-making.

Limitation 2: Sample Scope. We note that our findings are based on listed SOEsand recommend future studies to explore MOR effects in the context of non-listed SOEs, which represent a significant part of the state-owned sector.

Limitation 3: Contextual Factors. We mention that while we focused on regional marketization, future studies could investigate other moderating variables, such as industry characteristics or board composition, to build a more comprehensive contingency model.

**Comments（4）: Please re-check the English writing (grammar, spelling, sentences) and format at the possible final submission.**

**Response:** We sincerely thank the reviewer for this critical and constructive comment. We fully acknowledge the importance of linguistic accuracy and formatting consistency in ensuring the clarity, professionalism, and overall quality of the manuscript for final publication. We have taken comprehensive steps to address this point.

In direct response to this comment, we have undertaken a multi-layered approach to thoroughly polish the manuscript:

Comprehensive Professional Proofreading:We have conducted a line-by-line review of the entire manuscript, focusing on correcting grammatical errors, spelling mistakes, and improving sentence structure for better clarity and flow. Special attention was paid to technical terminology to ensure consistency and accuracy throughout the text.

Utilization of Editing Tools and Expertise:To ensure the highest standard of English, the revised manuscript has been checked using advanced grammar and spell-check software. Furthermore, it has been reviewed by a native English-speaking colleague with expertise in the field to refine the language and ensure it meets the standards for academic publication.
