# Supplementary material for: ‘Survival-driven’ or ‘policy-driven’—research on the motivation and economic consequences of the mixed-ownership reform of state-owned enterprises
Source: PLoS One. 2026 Jan 5;21(1):e0339970. doi: 10.1371/journal.pone.0339970 (PMC12768374; doi:10.1371/journal.pone.0339970)
Supplement: Attachment — Submitted filename: Response to Reviewers(2).docx [file pone.0339970.s006.docx]

Dear Editor of Plos ONE,

I sincerely appreciate the opportunity to revise our manuscript entitled "'Survival Needs' or' Policy Promotion '-- Research on the Motivation and Economic Consequences of the Mixed Ownership Reform of State owned Enterprises" . I am grateful to the reviewers for their insightful comments, which have significantly improved the quality of our work.Below is a summary of major revisions.

**Reviewer #3: After the author's revisions, the content of the paper has improved to some extent. However, further refinement is still needed in terms of linguistic aspects. Please conduct a thorough review of the entire text and polish it accordingly. If translation tools are used, ensure the sentence structures are adjusted to align with academic writing standards, avoiding excessive use of dashes.**

**Response：**To systematically optimize the language expression of the manuscript and ensure it fully meets the academic writing standards of international journals, we have taken targeted and rigorous improvement measures. Specifically, we commissioned the professional academic language editing team from American Journal Experts (AJE), a well-renowned institution in the field of academic editing, to conduct a comprehensive and meticulous revision of the entire text. The AJE team, with extensive experience in polishing academic papers in the financial field, has not only rigorously checked and standardized the vocabulary usage and grammatical accuracy of the manuscript but also focused on optimizing the sentence structures as you emphasized. Through such adjustments, the logical clarity and readability of the manuscript have been significantly enhanced.

Regarding your reminder about avoiding excessive use of dashes, we have carried out a sentence-by-sentence verification of the full text. For unnecessary or replaceable dashes, we have made appropriate revisions in strict accordance with academic writing conventions—such as replacing them with commas or semicolons, or restructuring the sentence structures—to ensure the formality and fluency of the manuscript are not affected by improper punctuation use.

To facilitate your review, we have attached the official editing certificate issued by the AJE team to this reply, which details the scope, standards, and completion status of the editing work.

**Reviewer #4: The manuscript is now well-written and can be considered for publication. But please do re-check the format and grammar.**

**Response：**To ensure the language accuracy and formatting standardization of the manuscript, we have taken rigorous and comprehensive measures. Specifically, we commissioned the professional academic language editing team from American Journal Experts (AJE) to conduct in-depth language polishing and thorough grammar checks on the entire text. The AJE team has carefully revised potential grammatical issues such as subject-verb agreement, tense consistency, and punctuation usage, and optimized the language expression to make it more in line with academic writing norms.

In addition to the language refinement by AJE, we have also conducted a systematic review of the manuscript format in strict accordance with the journal's author guidelines. We verified the consistency of citation styles, reference lists, table and figure captions, page layouts, and other formatting elements to ensure full compliance with the journal's requirements.

After the above series of language optimization efforts, we believe the professionalism and standardization of the manuscript’s language expression have been significantly enhanced. Here, we sincerely request that you review the revised manuscript once again. If there are any further issues regarding language or other aspects, please do not hesitate to point them out, and we will promptly make additional improvements.

Thank you for your patient guidance and generous support!

WangXiuting

2025.11.28
